# The Effect and Molecular Mechanism of *Fto* Gene Knockout on Cadmium-Induced Injury in Pancreatic β-Cells

**DOI:** 10.3390/cimb48040397

**Published:** 2026-04-13

**Authors:** Sina Yang, Wenhong Li, Shunrong Ma, Ning Xu, Kaiyan Shen, Jiamin Yuan, Yanying Hu, Shiyan Gu, Zuoshun He

**Affiliations:** Institute of Preventive Medicine, School of Public Health, Dali University, No. 22, Wanhua Road, Dali 671000, China

**Keywords:** cadmium, pancreatic β-cells, *Fto* gene, endoplasmic reticulum stress, oxidative damage

## Abstract

Cadmium exposure results in the impairment of pancreatic β-cells. The FTO protein, the product of the *Fto* gene, is a key regulator of diverse pathophysiological processes, including oxidative damage and cell death. However, it remains unclear whether *Fto* gene knockout affects cadmium-induced pancreatic β-cell damage, and the precise mechanisms involved are yet to be elucidated. Under conditions of cadmium exposure, *Fto* gene knockout was found to alleviate pancreatic β-cell damage significantly. Specifically, *Fto* gene knockout counteracted cadmium-induced cytotoxicity—manifested as reduced cell viability, increased apoptosis, and heightened lactate dehydrogenase (LDH) release—while simultaneously suppressing DNA damage and preserving cellular membrane integrity. On a molecular level, *Fto* gene knockout markedly mitigated cadmium-induced oxidative stress. This was achieved by curbing excessive reactive oxygen species (ROS) accumulation, lowering malondialdehyde (MDA) generation, and reducing 8-hydroxy-2′-deoxyguanosine (8-OHdG) levels, alongside restoring superoxide dismutase (SOD) activity. Furthermore, ER-Tracker Red staining revealed that cadmium treatment induced clustered aggregation of the endoplasmic reticulum (ER) and increased fluorescence intensity, suggesting the activation of endoplasmic reticulum stress (ERS). Conversely, *Fto* knockout ameliorated ER morphological abnormalities, thereby effectively antagonizing the excessive activation of ERS. In summary, our study elucidates the impact and underlying molecular mechanisms of the *Fto* gene in cadmium-induced toxicity in pancreatic β-cells from the perspectives of oxidative damage, ERS, and apoptosis. These findings identify the *Fto* gene as a potential molecular target for mitigating cadmium-induced toxicity in pancreatic β-cells, thereby providing a new theoretical basis for the prevention and treatment of cadmium-induced pancreatic β-cell injury.

## 1. Introduction

Cadmium (Cd), a prevalent environmental pollutant, can enter the human body through ingestion and inhalation, gradually accumulating and exerting toxic effects on various tissues and organs. Existing epidemiological evidence indicates that Cd exposure increases the risk of developing type 2 diabetes [1,2]. At the molecular level, studies have shown that Cd impairs the function of pancreatic β-cells through multiple pathways, including oxidative stress, endoplasmic reticulum stress (ERS), and apoptosis. This leads to impaired insulin secretion, thereby playing a significant role in the onset and progression of type 2 diabetes [3].

Cell injury is a complex biological process characterized by structural and functional abnormalities that occur when cells are exposed to stimuli from their internal or external environment. It is typically triggered by a variety of factors, including environmental stress, toxin exposure, and pathological conditions. These factors can disrupt normal cellular metabolism, function, and survival, leading to a cascade of adverse consequences. The manifestations of cell injury are diverse, with common types including cell death, apoptosis, oxidative damage, and ERS [4,5,6]. Oxidative injury stems from the excessive accumulation of reactive oxygen species (ROS). By inducing lipid peroxidation, protein carbonylation, and DNA strand breaks, ROS undermine cell membrane integrity, disrupt signal transduction, and induce genomic instability [7,8,9,10,11,12]. As the central site for protein synthesis and folding, the endoplasmic reticulum (ER) is highly sensitive to microenvironmental perturbations. Hypoxia, Ca^2+^ homeostasis imbalance, or oxidative stress can disrupt the oxidative folding microenvironment within the ER, leading to the accumulation of unfolded or misfolded proteins and subsequently triggering ERS [13,14,15]. To cope with this homeostatic disequilibrium, cells initiate the unfolded protein response (UPR) [16,17]. In its early stages, the UPR aims to restore homeostasis by attenuating protein translation, upregulating chaperone expression, and accelerating the degradation of misfolded proteins. However, when stress intensity exceeds the compensatory threshold or persists over time, the UPR shifts from an adaptive protective mechanism to a pro-apoptotic signaling pathway, ultimately driving the cell toward death [16,17,18,19,20,21,22]. Notably, a close bidirectional interaction exists between oxidative injury and ERS: ROS directly disrupts ER redox homeostasis, whereas persistent ERS amplifies ROS generation via mitochondrial pathways [23,24]. These processes are intertwined, forming a vicious cycle that collectively drives the pathological progression of neurodegenerative diseases, metabolic disorders, and cardiovascular diseases [24,25,26,27,28,29,30,31]. Therefore, elucidating the molecular regulatory mechanisms underlying this injury network is of great significance for clarifying the nature of diseases and developing novel targeted intervention strategies.

Numerous studies have confirmed that oxidative stress and ERS constitute core toxicological mechanisms mediating Cd-induced cellular injury [32,33,34,35]. Upon entering cells, Cd triggers an explosive accumulation of ROS and induces oxidative stress, primarily by displacing redox-active metal ions such as iron and copper and disrupting the mitochondrial electron transport chain [36,37,38]. Concurrently, Cd directly impairs the function of ER luminal chaperones (e.g., glucose-regulated protein 78 [GRP78]) and disrupts Ca^2+^ homeostasis, resulting in the excessive accumulation of misfolded proteins and the subsequent activation of the UPR [32,34,39]. In the context of Cd exposure, the sustained and aberrant activation of the UPR is often accompanied by the upregulation of pro-apoptotic signaling cascades, such as C/EBP homologous protein (CHOP) and caspase-12, ultimately culminating in apoptosis [34,40,41]. Consequently, systematically deciphering how Cd amplifies its cytotoxicity via the “oxidative stress-ERS” signaling network is critical for elucidating the pathogenesis of Cd-related diseases and identifying potential intervention targets.

In recent years, RNA epigenetic modifications—particularly the N^6^-methyladenosine (m^6^A) modification—have gained increasing recognition for their pivotal role in regulating responses to environmental stress [42,43]. As the first identified m^6^A demethylase, fat mass and obesity-associated protein (FTO) influences the fate of target transcripts by shaping their stability, splicing, and translation efficiency through the catalysis of m^6^A modification removal from mRNA, thereby participating in a wide range of physiological and pathological processes [43,44]. Emerging evidence indicates that FTO is deeply involved in the expression regulation of genes associated with oxidative stress and ERS. Through its demethylase activity, FTO exerts an indirect yet crucial regulatory effect on the expression of core antioxidant transcription factors [45,46] and key ERS molecules [47], thereby dynamically influencing the cellular stress tolerance threshold. For instance, in non-alcoholic fatty liver disease (NAFLD), elevated FTO expression is considered a key factor exacerbating hepatocyte injury, oxidative stress, and ERS, whereas inhibiting FTO effectively alleviates hepatocyte injury [48]. However, the specific role and molecular mechanisms of FTO in Cd-induced pancreatic β-cell injury, particularly whether it mediates downstream signaling events by regulating oxidative stress and ERS pathways, remain unclear.

This study employed an *Fto*-knockout pancreatic β-cell model; we systematically investigated the role of *Fto* in Cd-induced injury and its underlying molecular mechanisms. The results demonstrate that *Fto* knockout significantly inhibited the Cd-induced elevation of malondialdehyde (MDA), ROS, and 8-hydroxy-2′-deoxyguanosine (8-OHdG) levels, while simultaneously increasing superoxide dismutase (SOD) activity. Furthermore, it ameliorated ERS and effectively suppressed apoptosis, thereby playing a crucial protective role in Cd-induced pancreatic β-cell injury. Collectively, these findings expand the theoretical understanding of the molecular mechanisms underlying Cd-induced pancreatic β-cell injury and provide a novel theoretical basis for the prevention and treatment of Cd-induced diabetes.

## 2. Materials and Methods

### 2.1. Cell Culture and Treatment

In this study, we utilized wild-type mouse insulinoma cells (NIT1*^Wt^*) and *Fto*-knockout cells (NIT1*^Fto−/−^*) as experimental models, which were sourced from Shanghai Applied Protein Technology Co., Ltd. (Shanghai, China). The cells were routinely cultured in a humidified incubator at 37 °C with 5% CO_2_. The basal medium was DMEM/F-12 complete medium, supplemented with 9% fetal bovine serum (Wuhan Servicebio Technology Co., Ltd., Wuhan, China) and 1% penicillin-streptomycin (New Cell & Molecular Biotech Co., Ltd., Suzhou, China). Subculturing was performed when the cells reached 80% to 90% confluence [49].

### 2.2. Cell Viability Assessed by CCK-8 Assay

NIT1*^Wt^* and NIT1*^Fto−/−^* cells in the logarithmic growth phase were harvested and adjusted to a density of 8 × 10^4^ cells per well, then seeded into a 96-well plate at 100 µL per well. After cell attachment, the cultures were treated with CdCl_2_ (Sinopharm Group Chemical Reagent Co., Ltd., Shanghai, China) at final concentrations of 0, 0.4, 0.8, 1.6, 3.2, 6.4, 12.8, and 25.6 μmol/L, with three replicates per concentration, and incubated for 24 h. After treatment, the supernatant was removed, and each well was supplemented with 100 µL of serum-free medium containing 10% CCK-8 reagent [32]. The plate was incubated at 37 °C in the dark for 3.5 h. The absorbance (OD value) of each well was measured at a wavelength of 450 nm using a microplate reader (Multiskan GO, Thermo Fisher Scientific, Inc., Waltham, MA, USA). Cell viability was calculated as follows: Cell viability (%) = (OD of treated cells/OD of control cells) × 100%. For each biological replicate, the mean value of the three technical replicates was used for subsequent statistical analysis.

### 2.3. Detection of Apoptosis by TUNEL Assay

Cell apoptosis was detected using the one-step TUNEL cell apoptosis detection kit (Servicebio Technology Co., Ltd., Wuhan, China) according to the manufacturer’s instructions and a previously described method [50]. Cells were seeded at a density of 1 × 10^6^ cells/mL in 6-well plates containing pre-placed 14 mm sterile circular coverslips, with 1 mL of cell suspension per well. Following the designed treatments, the coverslips were gently rinsed twice with PBS. The cells were then fixed with 1 mL of 4% paraformaldehyde (PFA) solution for 15 min. After discarding the fixative, the cells were washed three times with phosphate-buffered saline (PBS) for 5 min each. For permeabilization, the coverslips were immersed in permeabilization solution and incubated at room temperature for 5 min. The solution was then removed, and the coverslips were washed three times again with PBS for 5 min each. Subsequently, 50 μL of Equilibration Buffer was added to each coverslip and incubated for 10 min at room temperature. After removing the buffer, 56 μL of TdT incubation buffer was added to each coverslip. The TdT incubation buffer was prepared by mixing recombinant TdT enzyme, TMR-5-dUTP labeling mix, and equilibration buffer at a ratio of 1:5:50 (1 μL:5 μL:50 μL). The coverslips were then incubated at 37 °C for 1 h. Following incubation, they were washed four times with PBS for 5 min each. Finally, an appropriate amount of DAPI solution was added to each coverslip. After incubating at room temperature for 8 min, the coverslips were washed three times with PBS for 5 min each. The coverslips were then mounted with an anti-fade mounting medium and observed under a fluorescence inverted microscope. For quantitative analysis, all fluorescence imaging experiments were performed with at least three independent biological replicates. In each replicate, at least five randomly selected, non-overlapping fields of view per sample group were captured. Image acquisition and subsequent ImageJ-based quantification of TUNEL-positive cells were carried out under strictly blinded conditions, meaning the operator was unaware of the sample group assignments during both image capturing and data analysis. Apoptotic cells were identified based on nuclear pyknosis observed by DAPI staining and positive TUNEL staining (red fluorescence). The apoptosis rate was calculated using the following formula: apoptosis rate (%) = (number of TUNEL-positive cells/total number of cells) × 100%.

### 2.4. Detection of ROS Levels Using Fluorescent Probes

Intracellular ROS levels were measured using the fluorescent probe DCFH-DA from Beyotime Biotechnology in Shanghai, China. After treatment according to the experimental design, the cell culture medium was removed. Then, 1 mL of 10 μmol/L DCFH-DA, prepared at a dilution ratio of 1:1000 in DMEM/F-12, was added to each well, and the cells were incubated at 37 °C in the dark for 20 min. Following incubation, the DCFH-DA working solution was aspirated, and the cells were washed three times with serum-free medium, with each wash lasting 5 min, to thoroughly remove any uninternalized probe [10]. All experiments were performed with at least three independent biological replicates. In each replicate, at least five randomly selected, non-overlapping fields of view per sample group were captured. Image acquisition and subsequent ImageJ-based quantitative analysis were carried out under strictly blinded conditions, i.e., the operator was unaware of the group allocation during both imaging and data analysis. Subsequently, random fields of view were selected for observation and imaging under a fluorescence microscope. Quantitative analysis of the images was conducted using ImageJ software version 1.52P from the National Institutes of Health in the USA. The mean fluorescence intensity of ROS was calculated using the following formula: mean fluorescence intensity = total optical density/area of analyzed cells.

### 2.5. Determination of MDA Levels and SOD Activity by Colorimetric Assay

Following protein extraction, the protein concentration was determined using a BCA protein assay kit (Beyotime Biotechnology, Shanghai, China). The intracellular levels of malondialdehyde (MDA) and the activity of superoxide dismutase (SOD) were measured using a lipid peroxidation (MDA) assay kit and a SOD activity assay kit (WST-8 method), respectively (both from Beyotime Biotechnology, Shanghai, China). The procedures were performed according to the manufacturer’s protocols and the method described by Li et al. [49]. Briefly, after the designed treatments, cells were collected and centrifuged. The supernatant was discarded, and the cell pellet was lysed in lysis buffer on ice for 30 min. The lysates were then centrifuged at 13,000 rpm (or 13,000× *g*) for 10 min at 4 °C. The resulting supernatant was collected for subsequent sample loading, incubation, and detection, following the instructions provided with each respective kit.

### 2.6. Assessment of Cell Death by AO/EB Dual Staining

To assess cell death, an acridine orange/ethidium bromide (AO/EB) dual staining assay was performed using an AO/EB staining kit (Maokang Biotechnology Co., Ltd., Shanghai, China). Cells were seeded into 6-well plates at a density of 1 × 10^6^ cells/well and incubated for 24 h at 37 °C in a 5% CO_2_ incubator to allow for complete attachment. The original medium was then discarded and replaced with fresh medium containing various concentrations of Cd chloride (0, 3.2, 6.4, and 12.8 μmol/L) for an additional 24 h of incubation. Following treatment, cells were collected by gently washing twice with pre-chilled PBS to remove residual medium. The cells were then resuspended in dilution buffer, and the cell density was adjusted to 1 × 10^6^ cells/mL. For staining, 2 μL of freshly prepared AO/EB working solution (AO:EB: dilution buffer = 1:1:8) was added to 25 μL of the cell suspension. The mixture was incubated for 10 min at room temperature in the dark [51]. Immediately after staining, the cells were observed under a fluorescence inverted microscope. All experiments were performed with at least three independent biological replicates. In each replicate, at least five randomly selected, non-overlapping fields of view per sample group were captured. The percentage of apoptotic cells was calculated as the ratio of apoptotic cells to the total number of cells counted (200 cells per sample).

### 2.7. Measurement of LDH Release by Colorimetric Assay

Cytotoxicity was assessed by measuring the amount of lactate dehydrogenase (LDH) released into the culture medium, using an LDH assay kit obtained from Nanjing Jiancheng Bioengineering Institute (Nanjing, China). Cells were seeded into a 96-well plate at a density of 8 × 10^4^ cells per well with 100 μL of cell suspension per well. After treatment according to the experimental design, the culture supernatant from each well was collected and centrifuged at 4000 rpm for 5 min [49]. The resulting supernatant was used for subsequent measurements. The assay was performed strictly in accordance with the kit instructions by adding the corresponding reagents sequentially. Following incubation, the absorbance of each well was measured at 450 nm using a microplate reader. LDH activity was calculated using the following formula: LDH activity (U/L) = [(OD_sample_ − OD_control_)/(OD_standard_ − OD_blank_)] × standard concentration × sample dilution factor × 1000.

### 2.8. Determination of 8-OHdG Levels by ELISA

The level of 8-hydroxy-2′-deoxyguanosine (8-OHdG) was measured using a competitive Enzyme-Linked Immunosorbent Assay (ELISA) with an 8-OHdG ELISA kit (Elabscience Biotechnology Co., Ltd., Wuhan, China), following the manufacturer’s instructions precisely. The procedure was as follows: First, cell lysates were centrifuged at 1000× *g* for 20 min at 4 °C. Then, 50 μL of the resulting supernatant was mixed with 50 μL of biotinylated antibody working solution and incubated at 37 °C for 45 min. The plate was washed three times with wash buffer. Subsequently, 100 μL of enzyme conjugate working solution was added, followed by a 15 min incubation period with 90 μL of substrate solution [52]. Finally, the reaction was terminated by adding 50 μL of stop solution, and the OD was measured at a wavelength of 450 nm. The concentration of 8-OHdG was calculated according to the instructions provided with the kit.

### 2.9. Visualization of Endoplasmic Reticulum Morphology Using Fluorescent Probes

Following the designated treatments, the original culture medium was removed, and the cells were gently washed twice with pre-warmed PBS at 37 °C to ensure complete removal of residual medium. To each well, 1 mL of ER-Tracker Red working solution (KeyGEN BioTECH Corp., Ltd., Nanjing, China) at a final concentration of 0.33 μmol/L was added. This working solution was prepared by diluting the ER-Tracker Red stock solution 1:3000 (*v*/*v*) in serum-free medium [53]. The cells were then incubated for 30 min in a 37 °C CO_2_ incubator (Revco Elite II, Thermo Fisher Scientific, Inc., Waltham, MA, USA). After incubation, the working solution was discarded, and the cells were washed three times with pre-warmed serum-free medium (5 min per wash). Subsequently, 1 mL of Hoechst 33,342 staining solution (Solarbio Science & Technology Co., Ltd., Beijing, China) was added to each well, and the cells were incubated for an additional 15 min at 37 °C in the dark. Following three washes with PBS, 1 mL of fresh serum-free medium was added. The cells were then incubated for 10 min at 37 °C in the dark to equilibrate the intracellular environment and reduce background fluorescence. All experiments were performed with at least three independent biological replicates. In each replicate, at least five randomly selected, non-overlapping fields of view per sample group were captured using a fluorescence microscope. Image acquisition and subsequent ImageJ-based quantitative analysis were carried out under strictly blinded conditions, i.e., the operator was unaware of the sample group assignments. The images were quantitatively analyzed using ImageJ software (version 1.52p, National Institutes of Health, Bethesda, MD, USA). The mean fluorescence intensity (MFI) of the ER was calculated using the following formula: MFI = integrated density/area.

### 2.10. Bioinformatics Analysis for Predicting Protein–Protein Interaction and m^6^A Modification Sites

Protein–protein interactions (PPIs) constitute the cornerstone of cellular functional networks. To systematically elucidate the mechanism of protein FTO in Cd-induced pancreatic β-cell injury and investigate its molecular function in ERS, we constructed its protein interaction network. Specifically, we first identified its core interacting proteins using the STRING database (v12.0). Subsequently, Cytoscape (v3.10.0) was employed to construct a global network and analyze the functional associations. Based on this network, we further focused on the ERS pathway, exploring the downstream signaling changes resulting from *Fto* gene deficiency by validating the specific interactions between FTO and core proteins. Furthermore, to explore the potential regulatory mechanisms of protein FTO at the epitranscriptomic level, this study also systematically predicted m^6^A modification sites on its transcripts. The full-length transcript sequences of the target gene were retrieved from the NCBI database (National Center for Biotechnology Information; https://www.ncbi.nlm.nih.gov/nucleotide/, accessed on 8 November 2025) and analyzed using the SRAMP online tool (https://bio.tools/sramp, accessed on 8 November 2025) for high-confidence site prediction.

### 2.11. Statistical Analysis

All data were obtained from at least three independent experiments and are presented as the mean ± standard deviation (SD). A one-way analysis of variance (ANOVA) was performed to compare differences among multiple groups. For post hoc comparisons between two groups, Tukey’s post hoc test was used when variances were equal, while the Kruskal–Wallis test was applied when variances were unequal. The correlation between variables was assessed using Pearson’s linear correlation analysis. Statistical analysis was conducted using SPSS software (version 27.0, IBM, Armonk, NY, USA). A *p*-value of less than 0.05 (*p* < 0.05) was considered to indicate a statistically significant difference.

## 3. Results

### 3.1. Effect of Cadmium on Cell Viability

**Figure 1:**
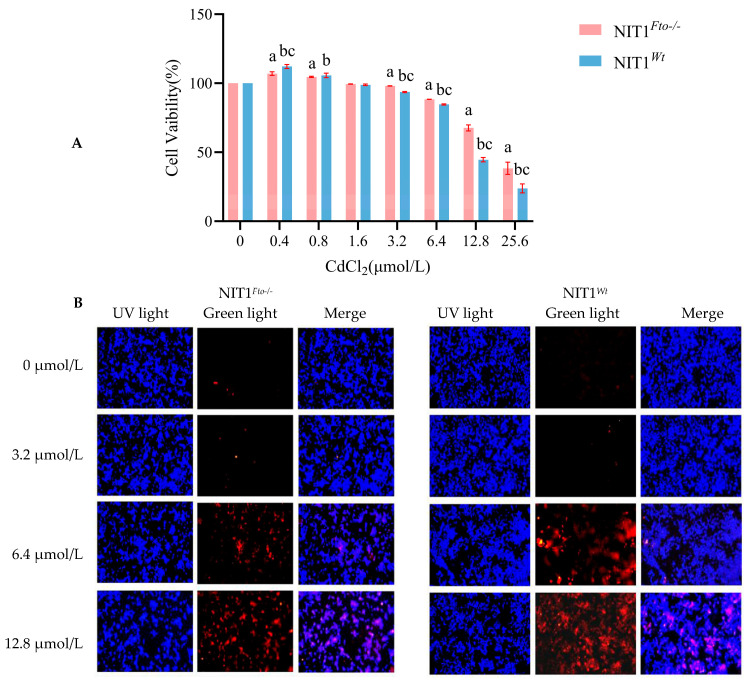

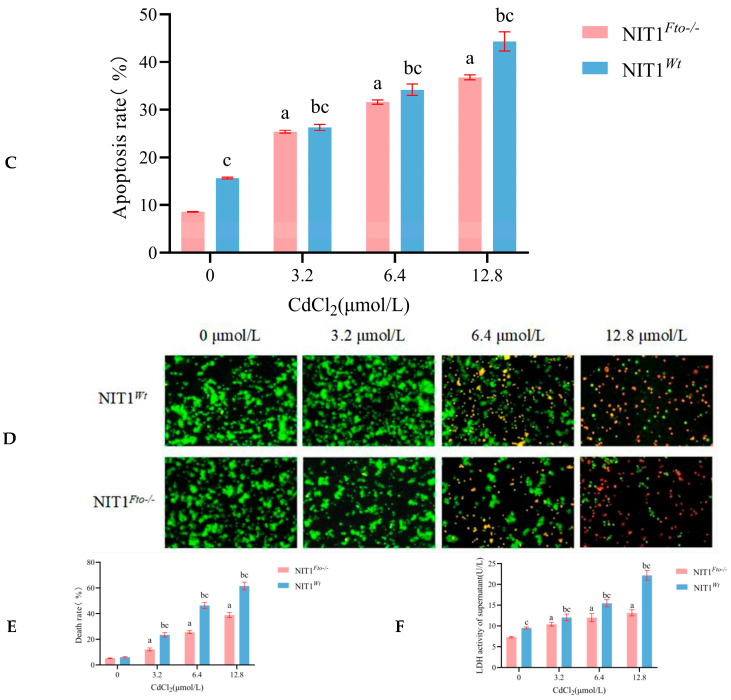
Effect of cadmium on cell death. Cells were treated with 3.2, 6.4, and 12.8 μmol/L of CdCl_2_ for 24 h, and the assays were performed according to the kit instructions. (**A**) Cell viability. (**B**) Representative images of cell apoptosis detected by the TUNEL assay (200×). Blue fluorescence indicates normal nuclei, while red fluorescence indicates apoptotic cells. (**C**) The apoptosis rate measured by the TUNEL assay. (**D**) Representative images of cell death detected by acridine orange/ethidium bromide (AO/EB) double staining (200×). Green fluorescence indicates live cells, while orange and red fluorescence indicate dead cells. (**E**) Percentage of dead cells detected by AO/EB double staining. (**F**) Lactate dehydrogenase (LDH) activity. “a” indicates a statistically significant difference compared to NIT1*^Fto−/−^* cells treated with 0 μmol/L of CdCl_2_ (*p* < 0.05); “b” indicates a statistically significant difference compared to NIT1*^Wt^* cells treated with 0 μmol/L of CdCl_2_ (*p* < 0.05); “c” indicates a statistically significant difference between NIT1*^Fto−/−^* and NIT1*^Wt^* cells exposed to the same concentration of CdCl_2_ (*p* < 0.05). All experiments were independently repeated three times.

As shown in Figure 1A, after treating NIT1*^Wt^* cells with a concentration gradient of Cd chloride (0, 0.4, 0.8, 1.6, 3.2, 6.4, 12.8, and 25.6 μmol/L), the cell viability rates were 100.00%, 113.94%, 104.56%, 98.69%, 93.06%, 84.22%, 45.01%, and 25.37%, respectively. In contrast, the viability rates in NIT1*^Fto−/−^* cells were 100.00%, 106.45%, 104.62%, 99.30%, 98.26%, 88.53%, 68.86%, and 40.34%, respectively. Notably, at treatment concentrations of 3.2, 6.4, 12.8, and 25.6 μmol/L, the viability of NIT1*^Fto−/−^* cells was significantly higher than that of NIT1*^Wt^* cells. Based on these results, we selected three concentrations—3.2, 6.4, and 12.8 μmol/L—for subsequent experiments.

### 3.2. Effect of Cadmium on Cell Apoptosis

As shown in Figure 1B, TUNEL-positive cells (red fluorescence) increased in a dose-dependent manner with increasing concentrations of CdCl_2_. The quantitative results (Figure 1C) indicated that the apoptosis rates in NIT1*^Wt^* cells treated with 3.2, 6.4, and 12.8 μmol/L CdCl_2_ were 26.30%, 34.19%, and 44.32%, respectively, which were significantly higher than that of the control group (15.66%). Similarly, under the same treatment conditions, the apoptosis rate in NIT1*^Fto−/−^* cells increased from 8.59% in the control group to 25.37%, 31.59%, and 36.79%, with all differences being statistically significant (*p* < 0.05). A similar trend was observed using AO/EB dual staining (Figure 1D). Compared to the 0 μmol/L group, the proportion of dead cells (orange and red fluorescence) in the CdCl_2_-treated groups (3.2, 6.4, and 12.8 μmol/L) gradually increased. The quantitative analysis (Figure 1E) revealed that the death rates in NIT1*^Wt^* cells were 23.47%, 46.33%, and 61.50%, respectively, significantly higher than the 0 μmol/L group (6.03%). Likewise, in NIT1*^Fto−/−^* cells, the death rate rose from 5.23% in the control group to 12.13%, 25.60%, and 38.87%, also with all differences being statistically significant (*p* < 0.05). In summary, under conditions of Cd exposure, the death rate of NIT1*^Wt^* cells was significantly higher than that of NIT1*^Fto−/−^* cells. This finding was further supported by the LDH release assay (Figure 1F). The results showed that LDH release from NIT1*^Wt^* cells increased in a concentration-dependent manner following treatment with various concentrations of CdCl_2_, measuring 9.51, 12.03, 15.45, and 22.16 U/L, respectively. Under the same conditions, LDH release from NIT1*^Fto−/−^* cells was 7.29, 10.42, 12.00, and 13.14 U/L, respectively. The differences between the two cell types were statistically significant at all tested concentrations (*p* < 0.05).

### 3.3. Effect of Cadmium on Cellular Oxidative Damage

**Figure 2:**
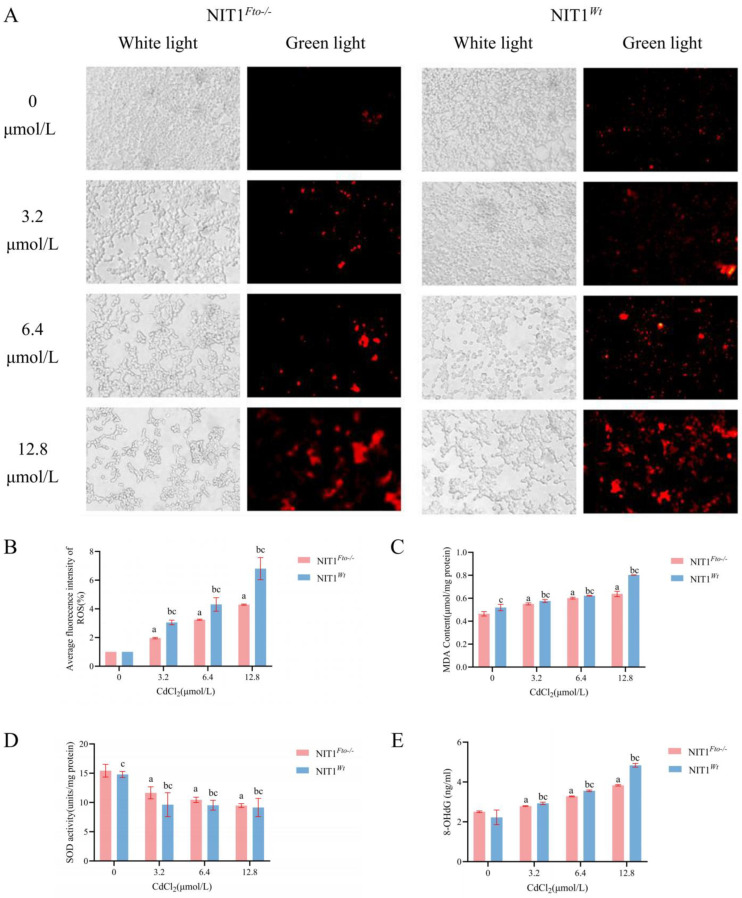
Effect of cadmium on indicators of cellular oxidative damage. Following treatment with different concentrations of CdCl_2_, the levels of ROS and MDA and the enzymatic activity of SOD were measured. (**A**) Representative images of reactive oxygen species (ROS) (200×), where red indicates ROS fluorescence. (**B**) Quantitative analysis of the mean fluorescence intensity of ROS. (**C**) Malondialdehyd (MDA) content. (**D**) Superoxide dismutase (SOD) activity. (**E**) 8-hydroxy-2’-deoxyguanosine (8-OHdG) levels. “a” indicates a statistically significant difference compared to NIT1*^Fto−/−^* cells treated with 0 μmol/L CdCl_2_ (*p* < 0.05); “b” indicates a statistically significant difference compared to NIT1*^Wt^* cells treated with 0 μmol/L CdCl_2_ (*p* < 0.05); “c” indicates a statistically significant difference between NIT1*^Fto−/−^* and NIT1*^Wt^* cells exposed to the same concentration of CdCl_2_ (*p* < 0.05).

As shown in Figure 2A, ROS detection results indicated a small amount of weak red fluorescence in the control group (0 μmol/L). With increasing concentrations of CdCl_2_ treatment, the red fluorescence intensity gradually intensified. The fluorescence signal was notably stronger in NIT1*^Wt^* cells compared to NIT1*^Fto−/−^* cells. Quantitative analysis (Figure 2B) revealed that in NIT1*^Wt^* cells treated with 3.2, 6.4, and 12.8 μmol/L of CdCl_2_, the mean ROS fluorescence intensities were 3.05, 4.31, and 6.80 times higher than the control, respectively. Under the same CdCl_2_ concentrations, NIT1*^Fto−/−^* cells exhibited mean ROS fluorescence intensities that were 1.96, 3.24, and 4.30 times that of the control. All treatment groups showed a statistically significant increase in mean fluorescence intensity compared to the control (*p* < 0.05). Furthermore, ROS levels were significantly higher in NIT1*^Wt^* cells than in NIT1*^Fto−/−^* cells across these concentrations (*p* < 0.05). As presented in Figure 2C, the MDA content in NIT1*^Wt^* cells increased significantly with the CdCl_2_ concentration, measuring 0.51, 0.57, 0.62, and 0.80 nmol/mg of protein, respectively. Similarly, a significant dose-dependent increase in the MDA content was observed in NIT1*^Fto−/−^* cells under the same treatment conditions, with values of 0.46, 0.55, 0.60, and 0.64 nmol/mg of protein (*p* < 0.05). Additionally, the MDA content was significantly higher in NIT1*^Wt^* cells than in NIT1*^Fto−/−^* cells at each corresponding concentration (*p* < 0.05). An opposing trend was observed for SOD activity. As the CdCl_2_ concentration increased, SOD activity in NIT1*^Wt^* cells showed a concentration-dependent decline, reaching values of 14.78, 9.62, 9.53, and 9.14 units/mg protein. A similar pattern was seen in NIT1*^Fto−/−^* cells, where SOD activity dropped significantly from 15.42 units/mg of protein to 11.65, 10.46, and 9.47 units/mg of protein. In both cell lines, all treatment groups differed significantly from their respective controls (*p* < 0.05) (Figure 2D). Notably, NIT1*^Wt^* cells consistently exhibited lower SOD activity than NIT1*^Fto−/−^* cells (*p* < 0.05). Consistent with these findings, the levels of 8-OhdG, a marker of oxidative DNA damage, were significantly elevated in CdCl_2_-treated NIT1*^Wt^* cells compared to the control group (*p* < 0.05). Specifically, 8-OhdG levels rose to 2.93, 3.56, and 4.84 ng/mL at CdCl_2_ concentrations of 3.2, 6.4, and 12.8 μmol/L, respectively, compared to the control level of 2.22 ng/mL. A similar, albeit less pronounced, increase was observed in NIT1*^Fto−/−^* cells, with 8-OhdG levels reaching 2.51, 2.79, 3.28, and 3.83 ng/mL, all of which were significantly different from the control (*p* < 0.05). Furthermore, 8-OhdG levels were significantly higher in NIT1*^Wt^* cells compared to their NIT1*^Fto−/−^* counterparts following Cd treatment.

### 3.4. Effect of Cadmium on the Intracellular Endoplasmic Reticulum

**Figure 3:**
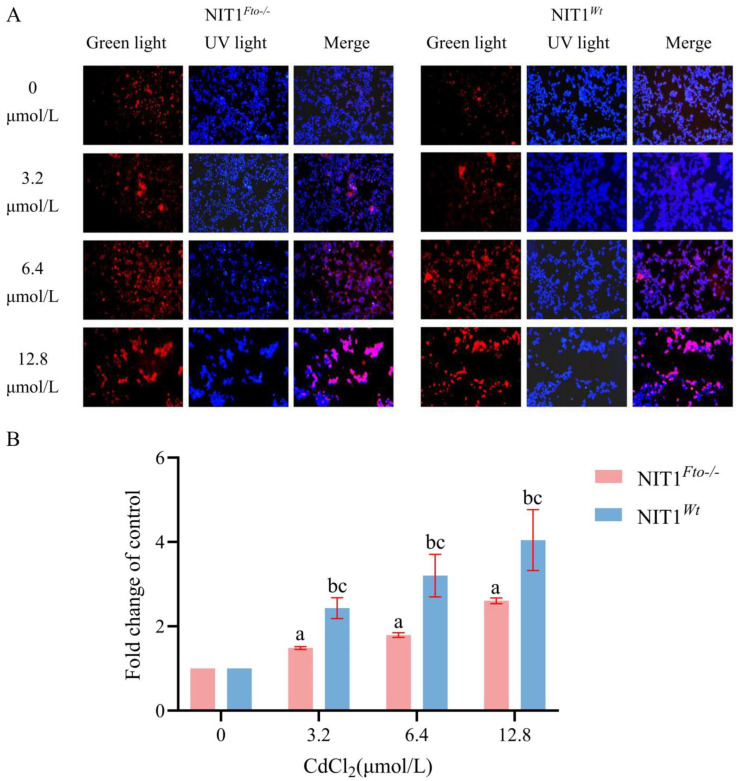
Effect of cadmium on changes in the endoplasmic reticulum. After treating cells with 0, 3.2, 6.4, and 12.8 μmol/L of CdCl_2_ for 24 h, they were stained with ER-Tracker Red to evaluate morphological changes in the endoplasmic reticulum. (**A**) Endoplasmic reticulum (ER) morphology and distribution in pancreatic β-cells (200×). Red indicates ER, and blue indicates nuclei. (**B**) Quantitative analysis of endoplasmic reticulum fluorescence intensity. “a” indicates a statistically significant difference compared to NIT1*^Fto−/−^* cells treated with 0 μmol/L of CdCl_2_ (*p* < 0.05); “b” indicates a statistically significant difference compared to NIT1*^Wt^* cells treated with 0 μmol/L of CdCl_2_ (*p* < 0.05); “c” indicates a statistically significant difference between NIT1*^Fto−/−^* and NIT1*^Wt^* cells exposed to the same concentration of CdCl_2_ (*p* < 0.05).

As shown in Figure 3A, ER-Tracker Red staining revealed that with increasing Cd concentrations, the ER underwent morphological changes characterized by clustered aggregation, accompanied by an increase in fluorescence intensity. Quantitative analysis further demonstrated that in NIT1*^Wt^* cells, the average fluorescence intensities of the 3.2, 6.4, and 12.8 μmol/L Cd chloride treatment groups were 2.43-, 3.20-, and 4.04-fold higher, respectively, than those of the control group (*p* < 0.05). Under the same conditions, the average ER fluorescence intensities in NIT1*^Fto−/−^* cells were 1.49-, 1.79-, and 2.61-fold higher than those in the control group (*p* < 0.05). Moreover, the enhancement in ER fluorescence intensity was concentration-dependent and significantly more pronounced in NIT1*^Wt^* cells compared to NIT1*^Fto−/−^* cells (*p* < 0.05).

### 3.5. Protein–Protein Interaction and m^6^A Modification Site Prediction

PPI network analysis revealed a close association between FTO and components of the ER protein processing pathway. Notably, WFS1 was identified as a direct and tightly interacting partner of FTO. A global connectivity analysis further revealed a functional module dominated by core hub proteins, including HSPA5 (connectivity = 106), HSP90B1 (connectivity = 77), and XBP1 (connectivity = 62). This module was tightly interconnected with factors such as DNAJC3 (connectivity = 59), ERN1 (connectivity = 48), ATF6 (connectivity = 55), ATF4 (connectivity = 27), and EIF2AK3 (connectivity = 26). FTO exhibited direct or indirect interactions with all the aforementioned hub proteins (highlighted as red and larger nodes in the figure). The formation of a dense interaction cluster with multiple neighboring proteins further established FTO’s central role within the network.

**Figure 4:**
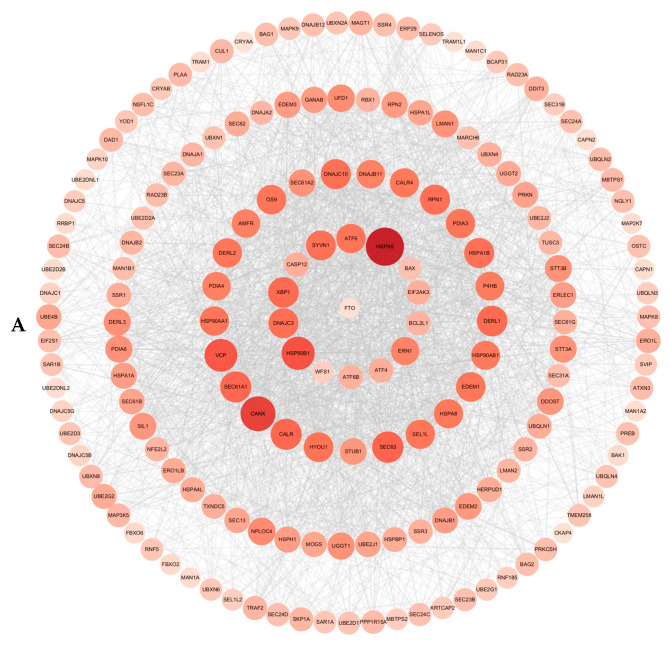

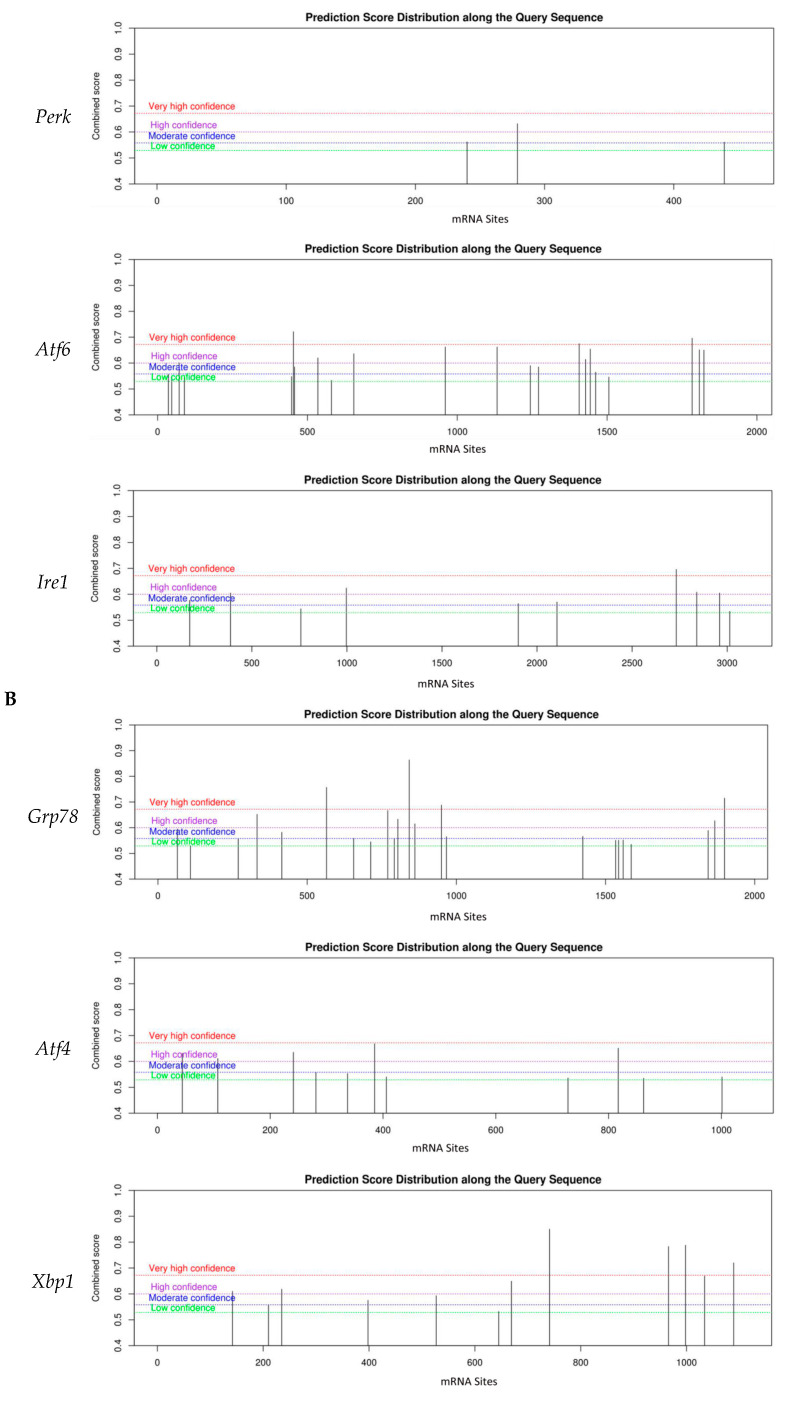

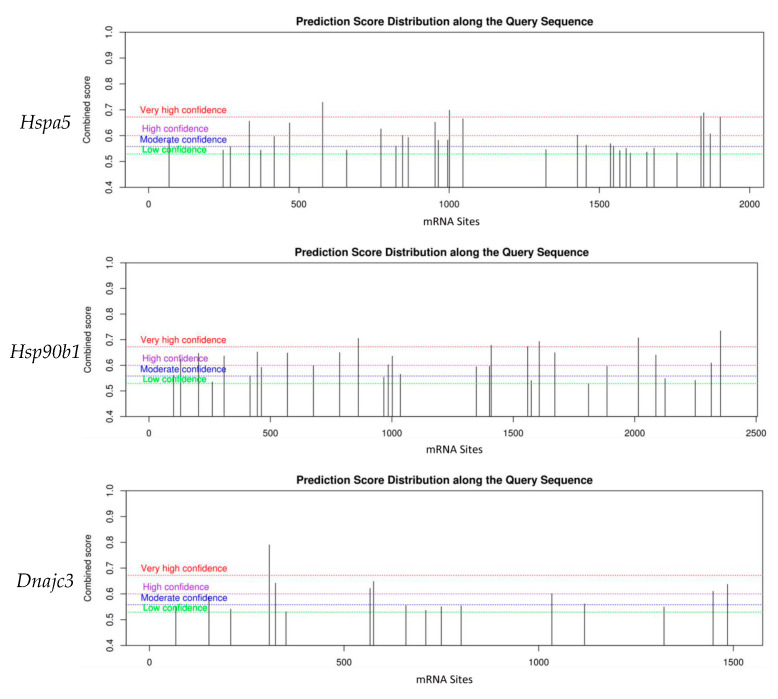
(**A**) Protein–protein interaction network. (**B**) Prediction of m^6^A modification sites.

Beyond protein–protein interactions, post-transcriptional modification is a key mechanism regulating protein expression. To investigate whether RNA-level modifications regulate the expression of these hub proteins, we systematically scanned the mRNA sequences of core ERS signaling molecules—*Perk* (*Eif2ak3*), *Atf6*, *Ire1* (*Ern1*), *Grp78* (*Hspa5*), *Atf4*, *Xbp1*, *Hsp90b1*, and *Dnajc3*—for m^6^A sites using the SRAMP tool. We found that transcripts of these key proteins commonly harbored multiple high-confidence or very-high-confidence m^6^A modification sites (Figure 4A). Notably, core regulatory factors such as *Atf6*, *Grp78*, *Xbp1*, and *Hsp90b1* exhibited a particularly high density of potential m^6^A modifications. Detailed information on site distribution and confidence scores is provided in Table 1.

## 4. Discussion

Cadmium is a ubiquitous environmental pollutant whose exposure is strongly correlated with an elevated risk of diabetes mellitus and impaired islet function [2,54,55]. Mechanistically, Cd interferes with the homeostasis of essential metals like zinc and calcium, which are vital for pancreatic β-cell function. This interference triggers ERS, ultimately leading to β-cell apoptosis [54]. Apoptosis is central to β-cell homeostasis, and its dysregulation is a critical driver of islet functional failure. Specifically, García-Aguilar et al. reported that in diabetes progression, β-cell death through apoptosis leads to decreased insulin secretion and functional loss, a process that involves ERS and mitochondrial dysfunction [56]. In recent years, the role of FTO-mediated m^6^A modification in Cd toxicity has gradually attracted attention. Studies in renal tubular epithelial cells have shown that Cd treatment not only induces elevated levels of oxidative stress but also significantly upregulates the expression of the *Fto* gene at both the mRNA and protein levels. Furthermore, recent research indicates that Cd exposure markedly promotes the expression of nuclear factor erythroid 2-related factor 2 (*Nrf2*) mRNA, and its expression is positively correlated with that of *Fto* and other m^6^A methyltransferases [57]. This suggests a potential functional association between the activation of the Nrf2 signaling pathway and FTO-mediated m^6^A modification. In bronchial epithelial cells, Cd exposure leads to significant cellular damage, characterized by decreased cell viability, increased oxidative stress, and enhanced apoptosis. Further mechanistic investigation revealed that FTO and its mediated m^6^A RNA modification play a crucial protective role in this process, significantly alleviating the Cd-induced cytotoxic effects [58]. However, research on whether FTO/m^6^A modification participates in Cd-induced pancreatic β-cell injury by regulating ERS remains very limited. Furthermore, the response of FTO to Cd toxicity may differ significantly across different tissues, which constitutes the core novelty that this study attempts to explore. In the context of this study, we observed an unexpected phenomenon: under treatment with the same concentration of CdCl_2_, the survival rate of NIT1*^Fto−/−^* cells was significantly higher than that of NIT1*^Wt^* cells. TUNEL and AO/EB staining confirmed that *Fto* knockout significantly reduced the Cd-induced apoptosis rate (Figure 1) and inhibited LDH release, which reflects cell membrane damage. This counterintuitive phenomenon indicates that in pancreatic β-cells, FTO does not play the traditional role of a “protector”; instead, its loss confers unexpected resistance to Cd toxicity. Regarding the intrinsic biochemical basis of this protective phenotype, previous studies have emphasized the complex role of FTO in regulating oxidative stress (e.g., via the pregnane X receptor (PXR)/*malat1* [59], circular RNA BRCA1 (*circBRCA1*)/forkhead box O1 (FOXO1) [32], and epigallocatechin-3-gallate (EGCG) degradation pathways [44]). In this study, we found that *Fto* knockout significantly inhibited the Cd-induced elevation of MDA, ROS, and 8-OHdG levels and enhanced SOD activity (Figure 2). This suggests that *Fto* knockout may greatly enhance the antioxidant defense capacity of pancreatic β-cells through epigenetic reprogramming.

Based on the classical theory that oxidative stress is a major upstream signal triggering ERS, we hypothesized that this potent antioxidant effect might block the signal transduction to downstream ERS at the source. Cd exposure can trigger ERS by activating the PERK-eIF2α-ATF4 pathway and inducing CHOP expression [35,60,61], while FTO can participate in ERS-mediated apoptosis via axes such as miR-503-5p/USP10 or by regulating lipid metabolism [47,51,62]. We observed that *Fto* knockout significantly inhibited Cd-induced ERS-related signals (Figure 3), corroborating this hypothesis. After establishing the phenotypic cascade linking *Fto* knockout to enhanced antioxidant defense and subsequent ERS inhibition, bioinformatics analysis further revealed its potential molecular basis. PPI networks demonstrated a significant interaction between FTO and components of the protein processing pathway in the ER; more critically, SRAMP database prediction indicated that the mRNAs of core regulatory genes in this pathway (e.g., *Grp78*, *Atf4*) generally contain high-confidence m^6^A modification sites (Figure 4). Based on these findings with our group’s previous discoveries, we propose the following hypothesis: *Fto* deficiency may alter the global m^6^A modification status, thereby enhancing antioxidant capacity to attenuate the initial triggering of ERS on one hand, and directly participating in the post-transcriptional expression of ERS core genes under Cd exposure as a potential regulatory node on the other. However, the aforementioned mechanistic hypothesis currently remains primarily at the level of correlation based on phenotypic observations and bioinformatics predictions, and a definitive causal link has not yet been established. Future studies are urgently needed to verify the presence of m^6^A modifications on these target genes using MeRIP-qPCR, and to thoroughly elucidate the m^6^A dependency and upstream-downstream driving relationships of this protective effect through *Fto* rescue experiments (including catalytically dead mutants) and specific ERS inhibitor interventions. Furthermore, this study currently lacks an assessment of protein levels for key ERS markers such as *p*-PERK, ATF6, and CHOP; moreover, the conclusions are based on a single in vitro cell model and fail to reflect the complex islet microenvironment in vivo. The completion of these functional validations and protein-level analyses, along with subsequent overall assessment of glucose homeostasis in animal models, will be the necessary extension to refine the mechanistic framework of this study.

## 5. Conclusions

**Figure 5:**
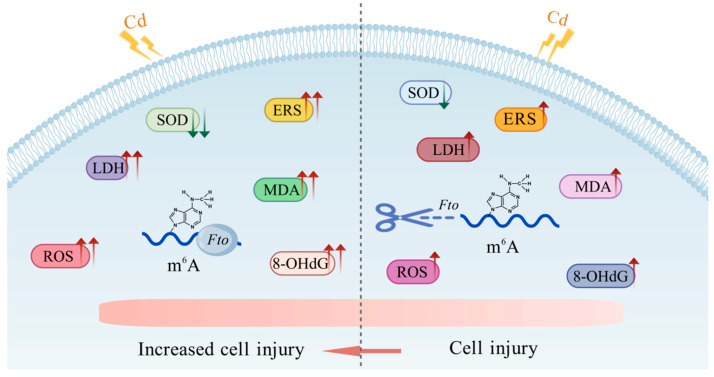
Proposed mechanism of *Fto* in cadmium-induced pancreatic β-cell injury (created with BioGDP.com [63]). Legend: ↑, upregulation/increase; ↑↑, significant upregulation/increase; ↓, downregulation/decrease; ↓↓, significant downregulation/decrease. Created with BioGDP.com. (2026). Agreement number: GDP2026XT0H20. https://BioGDP.com (accessed on 3 April 2026).

This study found that *Fto* knockout significantly alleviated CdCl_2_-induced apoptosis in pancreatic β-cells and reduced the overall cytotoxic effects of CdCl_2_. Mechanistically, we observed that *Fto* knockout effectively inhibited the excessive accumulation of ROS and attenuated ERS levels caused by Cd exposure (Figure 5). Furthermore, bioinformatics analysis predicted potential m^6^A modification sites on core ERS pathway genes (e.g., *Grp78* and *Atf4*). These findings establish a direct link between *Fto* deficiency and the anti-Cd phenotype in pancreatic β-cells, providing new data support for understanding epigenetic regulation under Cd exposure. Although the specific causal mechanisms between m^6^A modifications and target gene expression, as well as in vivo physiological effects, remain to be further validated, this study preliminarily establishes an association between FTO/m^6^A modifications and ERS in pancreatic β-cells, offering a promising entry point for in-depth exploration of the epigenetic mechanisms underlying Cd-induced type 2 diabetes.

## Figures and Tables

**Table 1:** Prediction of m^6^A modification sites in core endoplasmic reticulum stress genes.

Gene Name	Alternative Name (s)	Very-High-Confidence Sites (nt)	High-Confidence Sites (nt)
*Hspa5*	*Grp78/BiP*	579, 1001, 1838, 1847, 1902	335, 469, 773, 845, 953, 1046, 1427, 1869
*Hsp90b1*	*Grp94*	862, 1409, 1559, 1607, 2015, 2353	130, 204, 309, 446, 570
*Xbp1*	*-*	741, 966, 998, 1089	142, 235, 669, 1034
*Atf6*	*-*	453, 1407, 1784	72, 535, 655, 960, 1133, 1428, 1444, 1808, 1823
*Dnajc3*	*P58ipk*	308	324, 567, 576, 1034, 1448, 1485
*Eif2ak3*	*Perk*	-	279
*Ern1*	*Ire1*	2732	388, 997, 2480, 2960
*Atf4*	*-*	-	44, 107, 241, 385, 817

## Data Availability

The data of this study will be made available from the corresponding author upon reasonable request.

## References

[1] Zhou Y., Zhang J., Lei D., Qin Y., Chen Q., Tang N., Tang F., Huang S., Lu P., Jiang L. (2025). Association between heavy metal exposure and diabetic retinopathy related homeostatic dysregulation value in type 2 diabetic population: A cross-sectional study of NHANES 2003–2016. Ecotoxicol. Environ. Saf..

[2] Adokwe J.B., Pouyfung P., Kuraeiad S., Wongrith P., Inchai P., Yimthiang S., Satarug S., Khamphaya T. (2025). Concurrent lead and cadmium exposure among diabetics: A case-control study of socio-demographic and consumption behaviors. Nutrients.

[3] Mou Y., Sun Y., Liu G., Zhang N., He Z., Gu S. (2024). Screening of differentially expressed RNAs and identifying a ceRNA axis during cadmium-induced oxidative damage in pancreatic β cells. Sci. Rep..

[4] Liu S., Yao S., Yang H., Liu S., Wang Y. (2023). Autophagy: Regulator of cell death. Cell Death Dis..

[5] Long D., Mao C., Huang Y., Xu Y., Zhu Y. (2024). Ferroptosis in ulcerative colitis: Potential mechanisms and promising therapeutic targets. Biomed. Pharmacother..

[6] Zhang J., Guo J., Yang N., Huang Y., Hu T., Rao C. (2022). Endoplasmic reticulum stress-mediated cell death in liver injury. Cell Death Dis..

[7] Liang F.G., Zandkarimi F., Lee J., Axelrod J.L., Pekson R., Yoon Y., Stockwell B.R., Kitsis R.N. (2024). OPA1 promotes ferroptosis by augmenting mitochondrial ROS and suppressing an integrated stress response. Mol. Cell.

[8] Yin W., Xu H., Bai Z., Wu Y., Zhang Y., Liu R., Wang Z., Zhang B., Shen J., Zhang H. (2025). Inhibited peroxidase activity of peroxiredoxin 1 by palmitic acid exacerbates nonalcoholic steatohepatitis in Male mice. Nat. Commun..

[9] Yan J., Li Z., Liang Y., Yang C., Ou W., Mo H., Tang M., Chen D., Zhong C., Que D. (2023). Fucoxanthin alleviated myocardial ischemia and reperfusion injury through inhibition of ferroptosis via the NRF2 signaling pathway. Food Funct..

[10] Zhu M., Peng L., Huo S., Peng D., Gou J., Shi W., Tao J., Jiang T., Jiang Y., Wang Q. (2023). STAT3 signaling promotes cardiac injury by upregulating NCOA4-mediated ferritinophagy and ferroptosis in high-fat-diet fed mice. Free Radic. Biol. Med..

[11] Co H.K.C., Wu C.-C., Lee Y.-C., Chen S.-H. (2024). Emergence of large-scale cell death through ferroptotic trigger waves. Nature.

[12] Zhang H., Ma J., Hou C., Luo X., Zhu S., Peng Y., Peng C., Li P., Meng H., Xia Y. (2025). A ROS-mediated oxidation-O-GlcNAcylation cascade governs ferroptosis. Nat. Cell Biol..

[13] Christianson J.C., Jarosch E., Sommer T. (2023). Mechanisms of substrate processing during ER-associated protein degradation. Nat. Rev. Mol. Cell Biol..

[14] Ma M., Dubey R., Jen A., Pusapati G.V., Singal B., Shishkova E., Overmyer K.A., Cormier-Daire V., Fedry J., Aravind L. (2024). Regulated N-glycosylation controls chaperone function and receptor trafficking. Science.

[15] Hwang S.-M., Chang S., Rodriguez P.C., Cubillos-Ruiz J.R. (2025). Endoplasmic reticulum stress responses in anticancer immunity. Nat. Rev. Cancer.

[16] Acosta-Alvear D., Harnoss J.M., Walter P., Ashkenazi A. (2025). Homeostasis control in health and disease by the unfolded protein response. Nat. Rev. Mol. Cell Biol..

[17] Liu Y., Yang X., Zhou J., Yang H., Yang R., Zhu P., Zhou R., Wu T., Gao Y., Ye Z. (2024). OSGEP regulates islet β-cell function by modulating proinsulin translation and maintaining ER stress homeostasis in mice. Nat. Commun..

[18] Wong F., Li A., Omori S., Lach R.S., Nunez J., Ren Y., Brown S.P., Singhal V., Lyda B.R., Batjargal T. (2025). Optogenetics-enabled discovery of integrated stress response modulators. Cell.

[19] Wang S.-F., Tseng L.-M., Lee H.-C. (2023). Role of mitochondrial alterations in human cancer progression and cancer immunity. J. Biomed. Sci..

[20] Li W., Liu J., Yu T., Lu F., Miao Q., Meng X., Xiao W., Yang H., Zhang X. (2024). ZDHHC9-mediated bip/GRP78 S-palmitoylation inhibits unfolded protein response and promotes bladder cancer progression. Cancer Lett..

[21] Chaudhary M.R., Chaudhary S., Sharma Y., Singh T.A., Mishra A.K., Sharma S., Mehdi M.M. (2023). Aging, oxidative stress and degenerative diseases: Mechanisms, complications and emerging therapeutic strategies. Biogerontology.

[22] Wang Z., Tan C., Duan C., Wu J., Zhou D., Hou L., Qian W., Han C., Hou X. (2023). FUT2-dependent fucosylation of HYOU1 protects intestinal stem cells against inflammatory injury by regulating unfolded protein response. Redox Biol..

[23] Zhang S.X., Wang J.J., Starr C.R., Lee E.-J., Park K.S., Zhylkibayev A., Medina A., Lin J.H., Gorbatyuk M. (2024). The endoplasmic reticulum: Homeostasis and crosstalk in retinal health and disease. Prog. Retin. Eye Res..

[24] Morris H.E., Neves K.B., Nilsen M., Montezano A.C., MacLean M.R., Touyz R.M. (2023). Notch3/Hes5 induces vascular dysfunction in hypoxia-induced pulmonary hypertension through ER stress and redox-sensitive pathways. Hypertension.

[25] Camargo L.L., Wang Y., Rios F.J., McBride M., Montezano A.C., Touyz R.M. (2023). Oxidative stress and endoplasmic reticular stress interplay in the vasculopathy of hypertension. Can. J. Cardiol..

[26] Jiang R.-Q., Li Q.-Q., Sheng R. (2023). Mitochondria associated ER membranes and cerebral ischemia: Molecular mechanisms and therapeutic strategies. Pharmacol. Res..

[27] Zhang C., Lan X., Wang Q., Zheng Y., Cheng J., Han J., Li C., Cheng F., Wang X. (2025). Decoding ischemic stroke: Perspectives on the endoplasmic reticulum, mitochondria, and their crosstalk. Redox Biol..

[28] Lanzillotta C., Tramutola A., Lanzillotta S., Greco V., Pagnotta S., Sanchini C., Di Angelantonio S., Forte E., Rinaldo S., Paone A. (2024). Biliverdin reductase-a integrates insulin signaling with mitochondrial metabolism through phosphorylation of GSK3β. Redox Biol..

[29] Lei C., Lv Z., Ran Q., Jiang F., Zhang M. (2026). Homocysteine and diabetic retinopathy. Exp. Eye Res..

[30] Zhao J., Duan L., Li J., Yao C., Wang G., Mi J., Yu Y., Ding L., Zhao Y., Yan G. (2024). New insights into the interplay between autophagy, gut microbiota and insulin resistance in metabolic syndrome. Biomed. Pharmacother..

[31] Gancheva S., Roden M., Castera L. (2024). Diabetes as a risk factor for MASH progression. Diabetes Res. Clin. Pract..

[32] Zhang T., Sun S., Gavrilović A., Li D., Tang R. (2023). Selenium alleviates cadmium-induced oxidative stress, endoplasmic reticulum stress, and apoptosis in L8824 cells. Ecotoxicol. Environ. Saf..

[33] Huang Z., Xu R., Wan Z., Liu C., Li J., He J., Li L. (2025). Melatonin protects against cadmium-induced endoplasmic reticulum stress and ferroptosis through activating Nrf2/HO-1 signaling pathway in mice lung. Food Chem. Toxicol..

[34] Shi Y., Yan B., Huang R., Yu W., Xu B., Mao J., Liu Z., Wang J. (2025). Quercetin alleviated cadmium-induced oxidative stress, endoplasmic reticulum stress, and autophagy in chicken kidneys. Environ. Pollut..

[35] Huang D., Qiu M., Luo K., Zhu Y., Zhang S., He Z., Hu X., Cao Z. (2025). Puerarin prevents cadmium-induced endoplasmic reticulum stress via SIRT1-dependent PERK-CHOP pathway in HepG2 cells. Acta Biochim. Biophys. Sin..

[36] Jomova K., Alomar S.Y., Nepovimova E., Kuca K., Valko M. (2025). Heavy metals: Toxicity and human health effects. Arch. Toxicol..

[37] Arruebarrena M.A., Hawe C.T., Lee Y.M., Branco R.C. (2023). Mechanisms of cadmium neurotoxicity. Int. J. Mol. Sci..

[38] Rezaei K., Mastali G., Abbasgholinejad E., Bafrani M.A., Shahmohammadi A., Sadri Z., Zahed M.A. (2024). Cadmium neurotoxicity: Insights into behavioral effect and neurodegenerative diseases. Chemosphere.

[39] Mishra S., Paul R., Rani V., Ghosh D.K., Jain B.P. (2024). Cadmium toxicity on endoplasmic reticulum functioning. Int. J. Biochem. Mol. Biol..

[40] Cao Z., Tang C., Huang D., Zeng W., Han C., Li Z., Hu X. (2023). ROS-mediated PERK-CHOP pathway plays an important role in cadmium-induced HepG2 cells apoptosis. Environ. Toxicol..

[41] Zhang L., Zhang J., Zhou Y., Xia Q., Xie J., Zhu B., Wang Y., Yang Z., Li J. (2024). Azoramide ameliorates cadmium-induced cytotoxicity by inhibiting endoplasmic reticulum stress and suppressing oxidative stress. PeerJ.

[42] Wang J., Deng X., Jian T., Yin S., Chen L., Vergnes L., Li Z., Liu H., Lee R., Lim S.Y. (2025). DNA methyltransferase 1 modulates mitochondrial function through bridging m5C RNA methylation. Mol. Cell.

[43] Ponzetti M., Rucci N., Falone S. (2023). RNA methylation and cellular response to oxidative stress-promoting anticancer agents. Cell Cycle.

[44] Chen X., Wang Y., Wang J.-N., Zhang Y.-C., Zhang Y.-R., Sun R.-X., Qin B., Dai Y.-X., Zhu H.-J., Zhao J.-X. (2024). Lactylation-driven FTO targets CDK2 to aggravate microvascular anomalies in diabetic retinopathy. EMBO Mol. Med..

[45] Cheng Q., Zhou L., Fan X., Ma M., Zhang C., Zha X., Zhang Y. (2025). FTO-mediated Nrf2 demethylation alleviates high glucose-induced oxidative stress and apoptosis in retinal pigment epithelial cells. Mol. Biol. Rep..

[46] Gao X., Jia S., Gao L., Chen S., Zhang Y., Liang X., Zhang L., Zhang B., Meng C. (2025). MSC-derived exosomes alleviate oxidative stress-induced lysosomal membrane permeabilization damage in degenerated nucleus pulposus cells via promoting m6A demethylation of Nrf2. Free Radic. Biol. Med..

[47] Peng Q., Wang S., Huang S., Deng Y., Li Z., Liu C., Hong Y., Duan R., Xue X., Ge P. (2025). FTO/miR-503-5p/USP10 axis regulates neuronal endoplasmic reticulum stress-mediated apoptosis in ischemic stroke. Int. Immunopharmacol..

[48] Lim A., Zhou J., Sinha R.A., Singh B.K., Ghosh S., Lim K.-H., Chow P.K.-H., Woon E.C.Y., Yen P.M. (2016). Hepatic FTO expression is increased in NASH and its silencing attenuates palmitic acid-induced lipotoxicity. Biochem. Biophys. Res. Commun..

[49] Li W., Yang J., Zhao Y., Zhang N., Zhao B., Li R., Gu S., He Z. (2025). Cadmium treatment induces oxidative damage and apoptosis in vitro skeletal muscle cells. Toxicology.

[50] Wang J., Ding L., Wang K., Huang R., Yu W., Yan B., Wang H., Zhang C., Yang Z., Liu Z. (2022). Role of endoplasmic reticulum stress in cadmium-induced hepatocyte apoptosis and the protective effect of quercetin. Ecotoxicol. Environ. Saf..

[51] Chang K., Hong F., Liu H., Fang Y., Wang H., Song N., Ning Y., Lu Z., Jin S., Dai Y. (2025). FTO aggravates podocyte injury and diabetic nephropathy progression via m6A-dependent stabilization of ACC1 mRNA and promoting fatty acid metabolism. Biochem. Pharmacol..

[52] Spoto B., Politi C., Pizzini P., Parlongo R.M., Testa A., Mobrici M., Tripepi G.L., Mallamaci F., Zoccali C. (2025). 8-hydroxy-2′-deoxyguanosine, a biomarker of oxidative DNA injury, in diabetic kidney disease. Nutr. Metab. Cardiovasc. Dis..

[53] Xie H., Shi Y., Zhou Y., Liu H. (2022). TMBIM6 promotes diabetic tubular epithelial cell survival and albumin endocytosis by inhibiting the endoplasmic reticulum stress sensor, IRE1α. Mol. Biol. Rep..

[54] Liu J., Chen K., Tang M., Mu Q., Zhang S., Li J., Liao J., Jiang X., Wang C. (2025). Oxidative stress and inflammation mediate the adverse effects of cadmium exposure on all-cause and cause-specific mortality in patients with diabetes and prediabetes. Cardiovasc. Diabetol..

[55] Yu J., Wang C., Liu Y., Tao T., Yang L., Liu R., Liang D., Zhang Y., He Z., Sun Y. (2024). A comparative study of urinary levels of multiple metals and neurotransmitter correlations between GDM and T2DM populations. J. Trace Elem. Med. Biol..

[56] García-Aguilar A., Guillén C. (2022). Targeting pancreatic beta cell death in type 2 diabetes by polyphenols. Front. Endocrinol..

[57] Sun Y., Liu G., Li M., Wang L., He Z., Gu S. (2023). Study on the correlation between regulatory proteins of N6-methyladenosine and oxidative damage in cadmium-induced renal injury. Biol. Trace Elem. Res..

[58] Zhang N., Zhao Y., Yang J., Sun Y., Li R., He Z., Gu S. (2025). N6-methyladenosine mediated-NRF2 signaling pathway attenuates cadmium cytotoxicity by inhibiting oxidative damage in bronchial epithelial cells. Toxicol. Lett..

[59] Shao Y., Zhang Y., Zou S., Wang J., Li X., Qin M., Sun L., Yin W., Chang X., Wang S. (2024). (−)-epigallocatechin 3-gallate protects pancreatic β-cell against excessive autophagy-induced injury through promoting FTO degradation. Autophagy.

[60] Le Q.G., Ishiwata-Kimata Y., Kohno K., Kimata Y. (2016). Cadmium impairs protein folding in the endoplasmic reticulum and induces the unfolded protein response. FEMS Yeast Res..

[61] Li J.-Y., Cui D.-L., Xie Y.-M., Su J.-Z., Zhang M.-Y., Niu Y.-Y., Xiang P. (2022). Mechanisms of cd-induced cytotoxicity in normal human skin keratinocytes: Implication for human health. Int. J. Mol. Sci..

[62] Sun Y., Li R., Li W., Zhang N., Liu G., Zhao B., Mei Z., Gu S., He Z. (2024). Roles of m6A modification in regulating PPER pathway in cadmium-induced pancreatic β cell death. Ecotoxicol. Environ. Saf..

[63] Jiang S., Li H., Zhang L., Mu W., Zhang Y., Chen T., Wu J., Tang H., Zheng S., Liu Y. (2025). Generic Diagramming Platform (GDP): A comprehensive database of high-quality biomedical graphics. Nucleic Acids Res..

